# The Cost-effectiveness of Cefazolin Compared With Antistaphylococcal Penicillins for the Treatment of Methicillin-Sensitive *Staphylococcus aureus* Bacteremia

**DOI:** 10.1093/ofid/ofab476

**Published:** 2021-10-04

**Authors:** Elina Eleftheria Pliakos, Panayiotis D Ziakas, Eleftherios Mylonakis

**Affiliations:** Infectious Diseases Division, Warren Alpert Medical School of Brown University, Rhode Island Hospital, Providence, Rhode Island, USA

**Keywords:** *Staphylococcus aureus*, bacteremia, antistaphylococcal penicillins, cost-effectiveness

## Abstract

**Background:**

Methicillin-sensitive *Staphylococcus aureus* (MSSA) bacteremia is associated with significant morbidity, mortality, and hospitalization costs. Cefazolin and antistaphylococcal penicillins (ASPs), such as nafcillin, are the preferred treatments for MSSA bacteremia. The aim of this study was to compare the cost-effectiveness of each approach.

**Methods:**

We constructed a decision-analytic model comparing the use of cefazolin with ASPs for the treatment of MSSA bacteremia. Cost-effectiveness was determined by calculating deaths averted and incremental cost-effectiveness ratios (ICERs). Uncertainty was addressed by plotting cost-effectiveness planes and acceptability curves for various willingness-to-pay thresholds.

**Results:**

In the base-case analysis, the cost associated with the cefazolin strategy was $38 863.1, and the associated probability of survival was 0.91. For the ASP strategy, the cost was $48 578.8, and the probability of survival was 0.81. The incremental difference in cost between the 2 strategies was $9715.7, with hospital length of stay being the main driver of cost, and the incremental difference in effectiveness was 0.10. Overall, cefazolin results in savings of $97 156.8 per death averted (ICER, $–97 156.8/death averted). In the probabilistic analysis, at a willingness-to-pay of $50 000, cefazolin had a 68% chance of being cost-effective compared with ASPs. In cost-effectiveness acceptability curves, the cefazolin strategy was cost-effective in 73.5%–81.8% of simulations compared with ASP for a willingness-to-pay ranging up to $50 000.

**Conclusions:**

The use of cefazolin is a cost-effective strategy for the treatment of MSSA bacteremia and, when clinically appropriate, this strategy results in considerable health care cost-savings.

Bacteremia due to methicillin-sensitive *Staphylococcus aureus* (MSSA) bacteremia is associated with considerable morbidity, mortality, and hospitalization costs [1–4]. The rates of community-onset MSSA have increased at a rate of 3.9% per year between the years 2012–2017 [2] , and the costs associated with MSSA-related hospitalizations have converged with the costs of methicillin-resistant *S. aureus* (MRSA)–associated hospitalizations [4]. Optimizing antibiotic treatment for MSSA bacteremia is essential to reduce hospital costs, antibiotic resistance, and treatment-related adverse events [4–6].

MSSA bacteremia has traditionally been treated with antistaphylococcal penicillins (ASPs) such as nafcillin. Cefazolin is also an effective treatment strategy that is associated with less mortality [7–14] and fewer treatment-related adverse events [9, 10, 12, 13]. More specifically, cefazolin is associated with lower rates of nephrotoxicity and hepatotoxicity, with lower probability of discontinuation [15] and with more convenient dosing [16]. Given the current need for value-based decision-making [17, 18] that accounts for both health outcomes and health care expenditures, the aim of this study was to perform a cost-effectiveness analysis that compares the use of cefazolin with ASPs for the treatment of MSSA bacteremia.

## METHODS

### Model Structure

**Figure 1.**
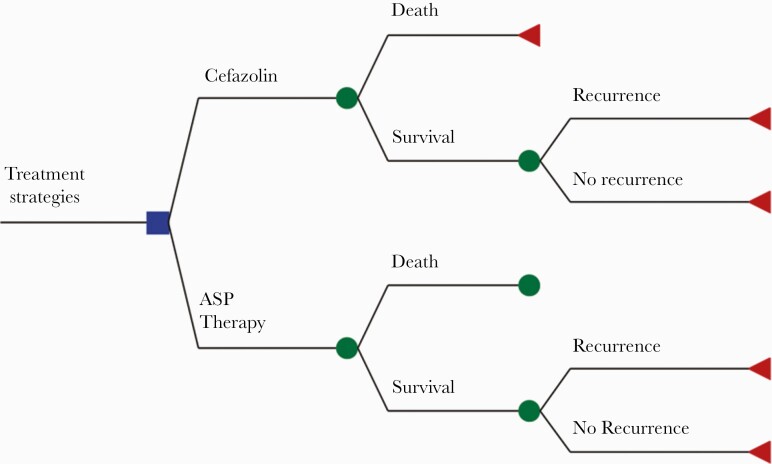
Decision tree model. The square indicates the decision to choose between the use of cefazolin or ASP therapy for the treatment of MSSA infection. The circles indicate chance nodes, and the triangles indicate end points. Abbreviations: ASP, antistaphylococcal penicillin; MSSA, methicillin-sensitive *Staphylococcus aureus*.

We constructed a decision model (Figure 1) assessing the cost-effectiveness of cefazolin compared with ASPs for the treatment of MSSA bacteremia. ASP therapy was defined as the use of intravenous oxacillin, nafcillin, or cloxacillin. Only nafcillin was considered in the determination of cost, as it is the ASP available in the United States [19]. The patient population of our analysis consisted of adult hospital in-patients with MSSA bacteremia. Costs and outcomes were calculated for a time horizon of 6 months, and the analysis was performed from a societal perspective.

Our study included an impact inventory, as recommended by guidelines [20]. The impact inventory is a checklist of health and nonhealth outcomes that were considered in this analysis, and it can be found in Table 1. Cost data were obtained from sources that reported values in US dollars. Mortality included 90-day mortality [7, 8, 10, 11, 14, 21]. Adverse events were defined as renal, hepatic, dermatological, or systemic [19], and MSSA-related readmission was defined as recurrence within 90 days [16]. The model was developed using the software TreeAge Pro 2019 (TreeAge, Williamstown, MA, USA).

**Table 1.** Impact Inventory

Sector	Type of Impact	Included in This Analysis From the Societal Perspective?	Notes on Sources of Evidence
	Formal health care sector		
Health	Health outcomes (effects)		
	Mortality	✓	See Methods
	Medical costs		
	Paid for by third-party payers	✓	
	Paid for by patients out-of-pocket	✓	
	Future related medical costs (payers and patients)	✓	
	Future unrelated medical costs (payers and patients)	✗	Not applicable
	Non–health care sector		
Productivity	Labor market earnings lost due to absence from work	✓	
	Uncompensated household production, patient	✗	

The analysis followed the recommendations made by the Consolidated Health Economic Evaluation Reporting Standards statement [22] and the guidelines reported in 2017 by the Second Panel on Cost-Effectiveness Analysis [20].

### Model Inputs: Assigning Probabilities

To identify studies that provide data on the effectiveness of cefazolin compared with ASP therapy for the treatment of MSSA, we used the most recently published (2014–2020) and relevant systematic reviews and meta-analyses [15, 16, 19, 23–25]. From these a 2019 meta-analysis by Lee et al. [19] was used as the basis of our analysis. The characteristics of the studies that were used to obtain effectiveness can be found in Supplementary Table 1. We included studies on MSSA bacteremia that compared cefazolin with any ASP that provided information on mortality. Probability estimates and confidence intervals for mortality, adverse events, and MSSA readmission rates were obtained by pooling the mortality, adverse event, and MSSA recurrence rates [7, 8, 10, 11, 14, 21] with the use of random-effects meta-analysis (Der Simonian and Laird; MedCalc, version 19.8) [26, 27]. This method was chosen as it accounts for the considerable interstudy differences and heterogeneity among the included studies [27].

### Model Inputs: Assigning Costs

Costs were obtained from the literature and were adjusted to January 2021 US dollars using the consumer price index inflation calculator provided by the Bureau of Labor Statistics [28]. The cost of ASP therapy per day was calculated as the average cost of nafcillin treatment per day, which was estimated at $225.0 [29], assuming that patients received the recommended dosing of 2 g intravenously every 4 hours [30]. Similarly, the cost of cefazolin per day was estimated to be $45.9 [31], assuming a dosing of 2 g intravenously every 8 hours [30]. The cost of preparing and administering the intravenous dose was estimated at $11.2 [32], resulting in a daily cost of $67.2 for nafcillin and $33.6 for cefazolin. Our model assumed that patients received antibiotic therapy for 14 days [30].

Hospitalization cost was estimated by multiplying the cost of hospitalization per day for the state of Rhode Island ($2964.4 adjusted), provided by the Kaiser Family Foundation [33], with the length of hospital stay for patients who received cefazolin (12 days) or ASP therapy (14 days) [13]. This cost of hospitalization was chosen because RI is the geographic base of our group and represents an average value for the various states across the United States [33]. The cost of lost productivity per day was estimated by multiplying the usual daily earning ($140.6) for US salary workers, provided by the US Department of Labor [34], by the length of stay associated with cefazolin or ASP therapy [13]. The average cost for a recurrence episode of MSSA was estimated to be $17 717.1 (adjusted) [35].

The base case costs of adverse events with nafcillin and cefazolin were estimated to be $2436.6 (adjusted) and $80.5 (adjusted), respectively. These were obtained from Flynt et al., who evaluated the adverse events between cefazolin and nafcillin for the treatment of MSSA bacteremia, using acute kidney injury as the primary study end point [13]. For the purposes of our analysis, these costs were multiplied by the probability of adverse events for each strategy [9, 10].

### Outcome and Data Analysis

In the base-case analysis, our primary outcome was the incremental cost-effectiveness ratio (ICER), defined as the ratio of the incremental cost between the 2 strategies (cefazolin or ASP therapy) over their incremental difference in effectiveness [22]. The incremental cost was defined as the excess cost of cefazolin therapy for the treatment of MSSA compared with the cost of ASP therapy. In turn, the incremental effectiveness was defined in terms of deaths averted.

The robustness of our model was evaluated with the use of deterministic (1-way sensitivity) and probabilistic sensitivity analysis (Monte Carlo). In the 1-way sensitivity analysis [36], each parameter was tested across a range of multiple point estimates, while in the probabilistic analysis we varied all parameters of the model simultaneously. The base-case estimates, ranges, and distributions for all parameters are presented in Table 2.

**Table 2.** Model Inputs and Baseline Estimates for Probabilities, Length of Stay, and Costs

	Base-Case Value (Range and Distribution)	Source
Probabilities		
Probability of mortality with cefazolin	0.09 (range, 0.03–0.18)	[7, 8, 10, 11, 14, 21]
	Uniform (0.03–0.18)	
Probability of mortality with ASP therapy	0.19 (range, 0.13–0.25)	[7, 8, 10, 11, 14, 21]
	Uniform (0.13–0.25)	
Probability of adverse events with cefazolin	0.08 (range, 0.02–0.19)	[9, 10]
	Uniform (0.02–0.19)	
Probability of adverse events with ASP therapy	0.30 (range, 0.07–0.36)	[9, 10]
	Uniform (0.07–0.36)	
Probability of recurrence of MSSA bacteremia with cefazolin	0.03 (range, 0.01–0.05)	[7, 9–11, 14]
	Uniform (0.01–0.05)	
Probability of recurrence of MSSA bacteremia with ASPs	0.02 (range, 0.01–0.04)	[7, 9–11, 14]
	Uniform (0.01–0.04)	
Length of stay or treatment, d		
Hospital length of stay with cefazolin	12 (range, 6–24)	[13]
	Gamma (12; SD, 3)	
Hospital length of stay with ASPs	14 (range, 7–28)	[13]
	Gamma (14; SD, 4)	
Days of treatment	14 (range, 7–28)	[8]
	Gamma (14; SD, 4)	
Costs, USD		
Cost of treatment with cefazolin per day	45.9 (range, 23.0–91.8)	[31]
	Gamma (45.9; SD, 11.5)	
Cost of treatment with ASP therapy per day	225.0 (range, 112.5–450.0)	[29]
	Gamma (225.0; SD, 56.3)	
Cost of IV preparation/administration for ASP per day	67.2 (range, 33.6–134.4)	[32]
	Gamma (67.2; SD, 16.8)	
Cost of IV preparation/administration for cefazolin per day	33.6 (range, 16.8–67.2)	[32]
	Gamma (33.6; SD, 8.4)	
Cost of hospitalization per day for the state of Rhode Island	2964.4 (range, 1482.0–5928.8)	[33]
	Gamma (2964.4; SD, 741.1)	
Cost of adverse events with cefazolin	80.5 (range, 40.3–161.0)	[13]
	Gamma (80.5; SD, 20.1)	
Cost of adverse events with ASPs	2436.6 (range, 1218.3–4873.2)	[13]
	Gamma (2436.6; SD, 609.2)	
Cost of lost productivity per day	140.6 (range, 70.3–281.2)	[13, 34]
	Gamma (140.6; SD, 35.2)	
Cost of MSSA recurrence episode	17 717.1 (range, 8858.6–35 434.2)	[35]
	Gamma (17 717.1; SD, 4429.3)	

Abbreviations: ASP, antistaphylococcal penicillin; IV, intravenous; MSSA, methicillin-sensitive *Staphylococcus aureus*.

Probabilities were modeled as uniform distributions (conservative modeling option), while costs were modeled as gamma distributions, as recommended by guidelines [37]. When a range was not available for a variable, we approximated it by allowing the variable to vary between 50% and 200% of its base case value [38]. If a standard deviation was not available, it was estimated by dividing the range by 6, as suggested for data that do not follow the normal distribution (approximation obtained with the use of Chebyshev’s inequality) [39].

In the Monte Carlo analysis [40], the model was run 10 000 times [38], and each time a value from the predetermined distributions (Table 2) was randomly selected for each variable. The results of each simulation were plotted on an incremental cost-effectiveness plane as points with coordinates (x,y), with x representing incremental effectiveness and y representing incremental cost. Points located within the southeast quadrant of the graph were considered to be cost-effective and dominant [41]. Finally, cost-effectiveness acceptability curves were used to evaluate the cost-effectiveness for various willingness-to-pay thresholds [42].

## RESULTS

In the base-case analysis, the cost for the cefazolin strategy was calculated to be $38 863.1, while the probability of survival was estimated to be 0.91. The cefazolin cost included the costs of medication, adverse events, hospitalization, lost productivity, and MSSA recurrence [7–11, 13, 14, 31, 33–35], while the probability for survival was estimated by pooling the available studies [7, 8, 10, 11, 14, 21]. Similarly, for the ASP strategy the cost was calculated to be $48 578.8 [7–11, 13, 14, 29, 33–35], and the probability of survival was 0.81 [7, 8, 10, 11, 14, 21].

The incremental difference in cost between the 2 strategies was $9715.7 ($38 863.1 vs $48 578.8), and the incremental difference in effectiveness was 0.10 (0.91 vs 0.81). Cefazolin prevented 1 death per 10 patients treated and resulted in savings of $97 156.8 per death averted (ICER, −$97 156.8 per death averted) compared with ASP therapy, suggesting that it is the more cost-effective strategy.

The sensitivity analysis, which allowed us to test each model variable for thresholds by varying each base-case value within the limits specified in Table 2, suggested that ASP therapy would become the cost-effective strategy if the length of stay with ASP therapy was shorter than 10.9 days or if the length of stay with cefazolin was >15.1 days. The findings of the sensitivity analysis are summarized in the tornado diagram (Figure 4), which is a graphical representation of how variations in each model variable affect the cost-effectiveness output.

**Figure 2.**
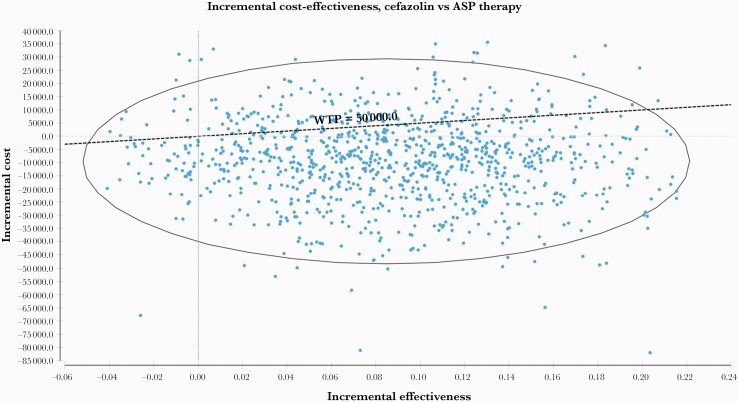
Incremental cost-effectiveness plane for cefazolin compared with ASP therapy. The y-axis represents incremental cost while the x-axis represents incremental effectiveness. Abbreviation: ASP, antistaphylococcal penicillin.

In the probabilistic analysis, the mean cost for cefazolin was estimated to be $38 715.2 (95% CI, $38 458.8–$38 971.7), and the mean cost for ASP therapy was estimated to be $48 188.1 (95% CI, $47 866.2–$48 509.9). In addition, in the cost-effectiveness plane (Figure 2), which aimed to show the uncertainty around the cost-effectiveness outcomes, cefazolin was located in the dominant and cost-effective quadrants in 68% of simulations.

**Figure 3.**
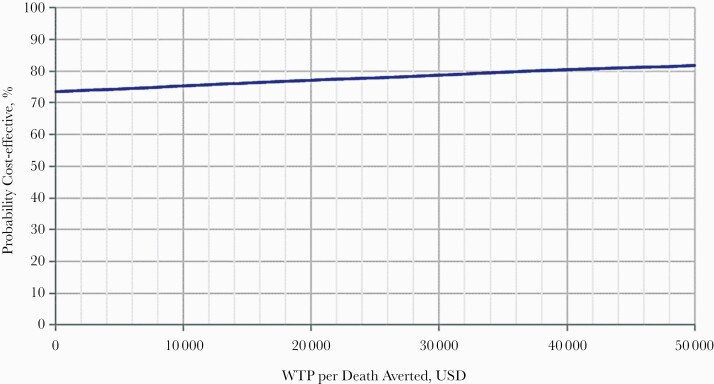
Cost-effectiveness acceptability curve with a willingness-to-pay ranging from $0 to $50 000. This curve shows the probability that cefazolin is a cost-effective strategy compared with ASP therapy, the baseline strategy, for a range of different cost-effectiveness thresholds. Abbreviation: ASP, antistaphylococcal penicillin.

Finally, in the cost-effectiveness acceptability curve (Figure 3), which shows the probability that cefazolin is cost-effective compared with ASP therapy for various willingness-to-pay thresholds, cefazolin was cost-effective in 73.5%–81.8% of simulations, for a willingness-to-pay ranging from $0 to $50 000.

## DISCUSSION

MSSA bacteremia is associated with high health care costs [4]. Cost-effectiveness analysis integrates information about health outcomes and health care expenditures [43] and can help inform value-based decision-making [17, 18]. Our study suggests that the use of cefazolin is a cost-effective strategy for the treatment of MSSA bacteremia compared with treatment with ASPs, as it prevented 1 death per 10 patients treated and resulted in savings of $97 156.8 per death averted (ICER, −$97 156.8 per death averted).

The fact that cefazolin is a cost-effective strategy can be explained from the lower mortality and lower rate of treatment-associated adverse events associated with this treatment option. For instance, a recent meta-analysis by Lee et al. that used data from 9 studies and 4442 patients estimated that cefazolin is associated with significantly lower rates of treatment failure, of crude all-cause mortality, and of treatment-related adverse events compared with ASP therapy [19]. Similarly, a retrospective cohort study conducted by Flynt et al. suggested that nafcillin is associated with a greater risk of nephrotoxicity compared with cefazolin, as nafcillin was an independent predictor of acute kidney injury [13].

Systematic reviews and meta-analysis studies have suggested that cefazolin should be preferred over ASPs as it associated with less mortality and has a better side effect profile [14–16, 19, 24, 44]. In terms of adverse events, a meta-analysis by Shi et al. that pooled data from 10 observational studies on the safety of ASPs vs cefazolin suggested that the safety of cefazolin is superior to ASPs particularly in terms of hepatotoxicity and nephrotoxicity [15]. The authors found no difference in safety with respect to risk of anaphylaxis and hematologic toxicity [15]. Similarly, a retrospective cohort analysis by Youngster et al. found that cefazolin has a better tolerability profile, as nafcillin treatment was associated with higher rates of both premature antimicrobial discontinuation (PAD) and drug-emergent events compared with cefazolin treatment [45]. In our study, mortality probabilities and adverse events were obtained by pooling data from a number of available studies [7–14]. Moreover, in clinical practice ASPs have been the standard of care over cefazolin due to concerns about the cefazolin inoculum effect [15]. This is a phenomenon that has been studied in vitro and refers to the loss of the therapeutic efficacy of cefazolin when large numbers of bacterial organisms are present [46]. Notably, the clinical relevance of the cefazolin inoculum effect seems to be limited and could be mitigated by an assay where ASP is used to decrease the bacterial load, followed by cefazolin for the majority of care [15, 16].

**Figure 4.**
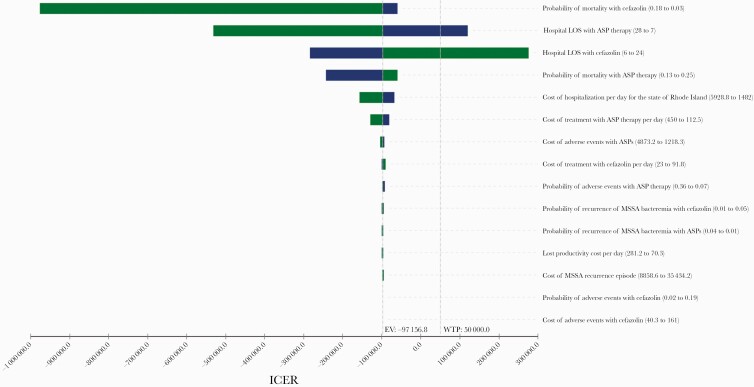
Tornado diagram. This graph is a summary of the 1-way sensitivity analysis. From top to bottom, it presents the variables that led to the greatest change in the ICERs. Green bars indicate that the ICER value decreases as the parameter value decreases, while the blue bars indicate that the ICER value increases as the parameter value increases. Abbreviations: EV, expected value; ICER, incremental cost-effectiveness ratio; LOS, length of stay; MSSA, methicillin-sensitive *Staphylococcus aureus*; WTP, willingness-to-pay.

Interestingly, our findings can be attributed to the fact that cefazolin was associated with a shorter hospital length of stay than ASPs. From the tornado diagram (Figure 4), it is evident that length of stay was the most important factor in determining cost-effectiveness findings. This is consistent with other studies suggesting that interventions that reduce the need for hospitalization are cost-effective [47–49]. Thampi et al., who performed a cost analysis using patients from 4 hospitals who were diagnosed with *S. aureus* bacteremia, pointed out that many programs may erroneously focus on antibiotic choice as the main driver of cost, when it is hospital length of stay and the intensity of care that mainly drive costs [3]. In addition, reduced length of stay can improve patient safety, patient outcomes, increase hospital bed capacity, and reduce costs [50]. Interestingly, it is possible that the observation that MSSA-related hospitalizations have converged with the costs of methicillin-resistant *S. aureus* (MRSA)–associated hospitalizations [4] could possibly be attributed to similar lengths of stay [3].

To perform our study, we conducted a comprehensive synthesis of data, but, as our study is a decision model, there are certain limitations that should be noted. Even though this study was conducted from a societal perspective and included an impact inventory that listed the health and nonhealth impact of interventions, there were limited data available on the costs of lost productivity and uncompensated household production that can result from MSSA bacteremia. Also, we did not distinguish between different types of ASPs as these were addressed together in the studies that we used to pool effectiveness estimates [7, 8, 10, 11, 14, 21]. Notably, the studies did not allow us to perform separate analyses based on the source of the bloodstream infection, and our model only takes MSSA bacteremia. More specifically, in high-burden infections, such as bone and joint infections, endocarditis, abscess, pneumonia, and epidural or intraspinal abscess, there is additional concern for treatment failure with cefazolin as these infections can overproduce type A beta-lactamase, which can hydrolyze cefazolin [19, 51, 52]. High-burden infections were included in the effectiveness data used in our study. However, specific cost data were not available, so we did not perform separate cost-effectiveness analyses. More information is needed for the modeling of deep-seeded infections such as osteomyelitis [16] and endocarditis [12, 15]. Similarly, there were limited data available on length of hospital stay and the cost of adverse events associated with each strategy. In order to address this lack of data, our sensitivity and probabilistic analyses have accounted for potential variations in the model inputs. Lastly, this study may not be generalizable to countries outside the United States, as nafcillin is the only ASP available in the United States and as the analysis was based on US cost values.

In conclusion, *S. aureus* has emerged as an urgent public health problem [53], and it is essential to identify effective and cost-effective treatment strategies. Our analysis combined data from multiple studies on the effectiveness of cefazolin compared with ASPs and indicated that cefazolin is a cost-effective strategy for the treatment of MSSA bacteremia. The low mortality, treatment-related adverse events, and length of stay associated with cefazolin seem to be driving this finding. Any treatment should always be tailored to individual patients, and our analysis provides useful information to clinical decision-makers about the economic benefit associated with using cefazolin over ASPs [54, 55]. A large randomized trial assessing mortality, length of stay, readmission, and the exact costs of MSSA bacteremia could provide an even more accurate estimate of the potential benefits associated with using cefazolin over ASPs. Furthermore, a stepwise approach where ASPs are given first, followed by transition to cefazolin for the remainder of treatment [16, 56], could be appropriate for some patients, especially those with high inoculum infections, and this approach should also be evaluated in future clinical trials.

## Supplementary Data

Supplementary materials are available at *Open Forum Infectious Diseases* online. Consisting of data provided by the authors to benefit the reader, the posted materials are not copyedited and are the sole responsibility of the authors, so questions or comments should be addressed to the corresponding author.

## Supplementary Material

ofab476_suppl_Supplementary_Materials
