# Supplementary material for: The Cost-effectiveness of Cefazolin Compared With Antistaphylococcal Penicillins for the Treatment of Methicillin-Sensitive Staphylococcus aureus Bacteremia
Source: Open Forum Infect Dis. 2021 Oct 4;8(11):ofab476. doi: 10.1093/ofid/ofab476 (PMC8566905; doi:10.1093/ofid/ofab476)
Supplement: ofab476_suppl_Supplementary_Materials [file ofab476_suppl_supplementary_materials.docx]

| Supplemental Table 1: Characteristics of Studies used to obtain base-case effectiveness estimates | | | | | | |
| --- | --- | --- | --- | --- | --- | --- |
| Study Author | **Study Design, Time Period, Country** | **Mean age (in years)** | **Gender (%male)** | **ASP studied** | **Cefazolin Total Population** | **ASP total population** |
| *S. Lee 2011* | Retrospective, case-controlled, single center, 2004-2006, Korea | cefazolin: 55  ASP: 52 | cefazolin:59  ASP:58 | nafcillin | 49 | 84 |
| *S. Pollett 2016* | Retrospective, cohort, single center, 2008-2013, USA | cefazolin: 53  nafcillin: 50 | cefazolin: 31 ASP: 23.3 | nafcillin | 70 | 30 |
| *J. Li 2014* | Retrospective, cohort, multicenter, 2008-2012, USA | cefazolin: 51  ASP: 51 | cefazolin: 75  ASP: 82 | oxacillin | 59 | 34 |
| *A. Bai 2015* | Retrospective, cohort, multicenter, 2007-2010, Canada | cefazolin: 68  ASP: 66 | cefazolin: 55  ASP: 67 | cloxacillin | 105 | 249 |
| *J. McDanel 2017* | Retrospective, cohort, multicenter, 2013-2015, USA | cefazolin: 64  ASP: 64 | cefazolin:97  ASP: 98.5 | nafcillin or oxacillin | 1163 | 2004 |
| *S. Lee 2017* | Prospective, cohort, multicenter, 2013-2015, Korea | cefazolin: 59  ASP: 63 | cefazolin: 61  ASP: 65 | nafcillin | 79 | 163 |
